# Special Issue: “Molecular Dynamics Simulations and Structural Analysis of Protein Domains”

**DOI:** 10.3390/ijms251910793

**Published:** 2024-10-08

**Authors:** Alexandre G. de Brevern

**Affiliations:** 1 DSIMB Bioinformatics Team, BIGR, INSERM, Université Paris Cité, F-75015 Paris, France; alexandre.debrevern@univ-paris-diderot.fr; Tel.: +33-1-4449-3000; 2 DSIMB Bioinformatics Team, BIGR, INSERM, Université de la Réunion, F-97715 Saint Denis, France

The 3D protein structure is the basis for all their biological functions. For more than 50 years, obtaining this structure has been difficult, costly, and sometimes impossible. Molecular modelling approaches are therefore making it possible to increase our knowledge by providing high-quality 3D structural models [1,2,3,4,5,6,7,8,9]. In this context, the AlphaFold2, AlphaFold3, ESMFold, and other deep learning approaches have enabled significant progress [10,11,12,13,14,15], with hundreds of millions of 3D structural models being made available to the scientific community [16,17,18].

However, the 3D structure gives only a small insight into biological function [14,19,20,21]. The 3D protein structure or 3D protein structural model is static, whereas the different parts of proteins are rigid, flexible, highly flexible, or even completely disordered [22,23,24,25]. Similarly, biological function can be associated with major conformational changes that are not provided by knowledge of a single structure [26,27]. It is therefore essential to understand the life of protein structures through their dynamics [28,29,30]. Molecular dynamics simulations (MDs), both classical and advanced, provide access to many more detailed questions of critical biomedical interest [31,32,33,34,35,36]. For example, a point mutation in a structural model does not explain the functional change at the atomic level. MD simulations are needed to generate hypotheses about the effects of the mutation [37,38,39,40,41]. In this way, MDs allow us to see allosteric changes that are sometimes impossible to characterise experimentally [42,43,44,45,46,47].

This Special Issue had so focussed on issues ranging from the most fundamental to the most applied research on protein structures (or structural models) through the use and/or development of both classical and more innovative simulations. In the end, eight articles were accepted for publication in this Special Issue. I would like to thank the authors for their confidence at the time of submission and also those who unfortunately did not make it to the end of the process. I would also like to mention the involvement of several other editors specialising in this area, including Istvan Simon, Paulino Gómez-Puertas, Stefanie Krick, and Yuri Lyubchenko, as well as the administrative staff who ensured that the review process ran smoothly. The articles deal with very different systems and approaches, which have similarities but often very different aims. The five research articles are presented first, followed by the three review articles.

To begin with, López-Luis, Soriano-Pérez, Parada-Fabián, Torres, Maldonado-Rodríguez, and Méndez-Tenorio propose a work that may seem simple: a structural model [48]. Their work concerns the largest protein of *Helicobacter pylori* (Hp), a bacterium detected in the stomach of more than 50% of the human population [49]. In some well-documented cases, Hp can cause gastroduodenal ulcers and become a risk factor for gastric cancer (GC) [50,51,52]. These strains encode virulence genes such as type IV secretion system (T4SS), adhesins, and a cytotoxin [53]. The largest protein of the Hp T4SS is CagY (a little less than 2000 amino acids). Only its carboxyl C-terminal region has known homology; the rest have no simple direct links to structural homologues [54]. They split the protein into seven main domains, starting by modelling the C-terminal part, which has a structural support [54]. They use different approaches, such as I-TASSER [6], ROSETTA [8], and combine the results with different structural supports using SWISSMODEL [55]. For the other regions, they repeat similar approaches, adding AlphaFold/CollabFold [10,55] and monitoring the results. An important point emphasised is the fact that only dimers can be approached due to computational limitations. The models are relaxed by short MDs. Classical evaluation approaches, such as ProSA-web [56], ERRAT [57], and QMEANDisCo [58], are also used to select the best models. The final model is obtained by adjusting the constraints imposed by the disulphide bridges between the different obtained chains. The article is very methodological, comparing models from different tools and confronting them with experimental data [48]. Figure 1 highlights the difficulty of the task as well as the beauty of the overall topology of the complex.

Ziada, Diharce, Serillon, Bonnet, and Aci-Sèche have worked on a specific human cyclin-dependent kinase (CDK) called CDK8 [60]. CDKs are a family of serine-threonine kinases that need to bind to regulatory proteins called cyclins in order to be active. CDKs are key regulators of the cell cycle and gene transcription [61]. CDK8 is an interesting target that has recently attracted considerable attention following the publication of numerous genetic and biochemical studies highlighting its many key roles in oncogenesis [62,63,64]. The activity of CDK8 is controlled by a regulatory protein called cyclin C (CycC). MD simulations with binding free energy calculations allowed analysing the effect of CycC binding on the structure and dynamics of CDK8, as shown in Figure 2. The authors were able to simulate the transition between the active and inactive forms. CycC has a stabilising effect on CDK8, highlighting specific interaction hotspots in its interaction surface compared to other human CDK/Cyc pairs [60]. These results underline the importance of retaining CycC in computational studies when studying the human CDK8 protein in both its active and inactive forms.

Botnari and Tchertanov have focused on human vitamin K epoxide reductase (hVKORC1), an endoplasmic reticulum (ER) protein that reduces inactive vitamin K 2,3-epoxide to active vitamin K quinone, i.e., a protein required for blood coagulation [65]. Structurally, hVKORC1 is a small modular protein with a transmembrane domain, an intrinsically disordered L-loop that projects into the ER, and highly flexible N- and C-terminals that float in the cytoplasm. Genetic polymorphisms of hVKORC1 are associated with low or accelerated rates of vitamin K recycling, leading to serious conditions such as bleeding and thrombosis. Surprisingly, the hVKORC1 polymorphism affects the response to doses of anti-vitamin K anticoagulants, promoting resistance to treatment. These studies follow previous studies made by the same group [66,67,68]. They used a de novo model [69] to perform MD simulations of the wild-type hVKORC1 and four different pathological mutants. All proteins have similar topology but different dynamics. L-loop missense mutations affect the folding and dynamic properties of the L-loop itself as well as the transmembrane domain. The extended ‘open’ conformation is either rarely observed (hVKORC1^A41S^ and hVKORC1^W59R^) or never observed (hVKORC1^S52W^ and hVKORC1^H68Y^). The compact globular conformation of the L-loop is mainly maintained by van der Waals contacts, specific for each mutant. These studies make it possible to define specific pockets during dynamics in each of these systems (see Figure 3) [65].

Delort, Cottone, Malliavin, and Müller attack a membrane system of biomedical interest. Botulinum neurotoxins (BoNT) [70], secreted by *Clostridium botulinum*, are among the most potent toxic compounds in the world, causing flaccid paralysis in the host [71,72]. The toxins consist of two protein chains linked by a disulphide bridge: the light chain (LC) and the heavy chain (HC). The role of the HC is to prepare the way for the catalytic region of the light chain, which is responsible for the toxicity of BoNT [73,74]. The system is complex because it is highly sensitive to pH, and especially in the dimeric structure, the switching domain changes from *α*-helices to *β*-strands, a quite drastic evolution [75]. The work by Delort and colleagues studies the internal dynamics of the translocation domain in water and in a mixture of water and ethanol using MDs over microseconds. A combination of elasticity theory and geometry is used to describe the different configurations of the system. A very specific feature of the work presented is the use of theoretical mechanisms known as the Twister mechanism and the Darboux torque mechanism [76], which make it possible to understand how membrane deformation is induced. They have been successfully applied in [77,78,79,80]. This study is the first to link the Twister mechanism with biological interactions.

Another specific feature of this research is the work carried out in different media, such as ethanol (see Figure 4) [70]. This rather complex approach represents the first step in coupling the mesoscopic Twister model to an atomistic approach with predictive power for protein–membrane interactions. By using the two approaches together, it has been possible to propose a hierarchy of interactions between the translocation domain and the membrane. This results in a partitioning of the protein structure into regions undergoing unfolding or separation from the protein core, and helix bundles 1 and 2 as the main players involved in insertion into the membrane.

Tam, Qin, Zhao, Sinha, Lei, and Wang focus on a current issue, the impact of variants on possible pathogenicity [82]. Indeed, databases often give a benign, probably benign, pathogenic, or probably pathogenic association for a given variant, but also variants of uncertain significance (VUS) due to the lack of functional evidence. Their number is far from negligible, which makes the analyses and predictions of doctors particularly complex. They are never integrated into pathogenicity prediction methods or their evaluations [83,84,85,86,87,88,89]. Lam and their colleagues focused on a specific case: the DNA mismatch repair (MMR) gene MLH1 is associated with Lynch syndrome (LS), an autosomal dominant inherited cancer [90,91]. Indeed, more than 80% of the data on this gene are associated with VUs. Of particular interest, the authors have developed a structure-based method called deep learning-Ramachandran plot-molecular dynamics simulation (DL-RP-MDS) [92]. This method is designed to assess the harmfulness of VUS. In practical terms, the method extracts structural information from proteins using the Ramachandran plot-molecular dynamics simulation (RP-MDS) method [93]. Thus, a point of importance is the work carried out from the Ramachandran plot data [94], which has been evolving since 1963 but remains essential in our fields [95,96,97,98]. The crystal structure of MLH1 (PBD ID: 4P7A [99]) was used. The mutant structures for each missense variant were also built following classical procedures [23] with MODELLER [4] and best model selection by zDOPE [100]. Long MDs were performed and analysed. In a second step, the variation data are combined with an unsupervised learning model consisting of an autoencoder and a neural network classifier to identify variants that cause a significant change in protein structure. They applied the method to classify 447 MLH1 missense VUSs and predicted that 28.2% of MLH1 missense VUSs were deleterious. This approach allows us to prioritise costly experiments by searching for the most likely cases [82]. A major interest of the approach is the link with the structure that allows, as represented in Figure 5, to understand the atomic mechanism associated with a variant.

Qvit and colleagues then provide us with two interesting reviews on protein kinase C (PKC). Indeed, protein kinases are one of the most important drug targets in the human proteome, mainly due to the treatment of cancer, cardiovascular diseases, and a growing number of other diseases, including autoimmune and inflammatory processes. In a first review, Silnitsky, Rubin, Zerihun, and Qvit review the role of different PKC isoforms in cancer and cardiovascular diseases, with a particular focus on PKC family inhibitors [101]. They discuss translational examples and carefully examine the advantages and limitations of each compound. The review first briefly introduces kinases [102,103,104,105,106] and then focusses on the protein kinase C family [107,108,109] (see Figure 6). It highlights its regulation by lipid second messengers [110,111] and its regulations by scaffold interactions [112,113,114], and shows its implications in cancers [106,115,116,117,118], in cardiovascular diseases [119,120,121,122,123], and other important human diseases [124,125,126,127]. It ends with how to target PKC with its PKC inhibitors in clinical trials [101,128,129,130,131,132,133].

In the second review, Zerihun, Rubin, Silnitsky, and Qvit focus on peptides as allosteric modulators of protein kinase C targeting protein–protein interactions [134]. They are investigating alternative allosteric binding mechanisms to target PKC and new drug platforms, in particular modified peptides. The idea is to design protein kinase modulators with increased selectivity and improved pharmacological properties. In this context, molecular docking to predict the mechanisms of action of inhibitor-kinase interactions can greatly facilitate the development of next-generation PKC modulators. Pleasingly, they provide structural information on protein kinases [106,135], with protein kinases as major drug targets [136], the concept of non-catalytic domains of protein kinases [137], allosteric modulation of kinases [138], the use of allosteric sites in drug discovery [139], allosteric regulation by protein–protein interactions [140], and then move on to peptides targeting protein–protein interactions [141]. They describe the therapeutic use of peptides and approaches to developing peptides as regulators of protein kinases targeting allosteric sites [142]. They then present peptides derived from unique substrate sites [143,144] together with peptides derived from the pseudo-substrate site [145,146], those derived from substrate phosphorylation sites [147], and those derived from substrate protein–protein interaction sites [148]. Authors underline these examples by their own work on a selective inhibitor of mitofusin 1-βIIPKC associations that improves heart failure outcome in rats [149]. Figure 7 shows the principle of the dedicated approach.

These results then raise the more complex issue of peptides derived from similar sequences in binding proteins. Indeed, in some cases, signalling enzymes interact with several proteins that are unrelated to each other [150]. In many cases, these unrelated proteins share a short sequence of homology that could represent the enzyme-binding site [151]. They discuss peptides derived from sequences involved in intramolecular interactions [152]. Following these examples, using advanced computational biology approaches, they highlight evolutionarily conserved peptides and, again, complex examples of peptides derived from conserved sequences in homologous but unrelated domains of proteins [153,154]. To limit the possibility of cross-reactions, peptides derived from unique protein kinase sequences have been developed, sometimes even targeting a specific isoform [155].

Finally, Zheng, and Liu conclude the Special Issue with a new view on the study of Fos-related antigen-2 (Fra-2) in respiratory diseases [156]. Fra-2 is a member of the AP-1 (activator protein 1) family of transcription factors. It is involved in the control of cell growth and differentiation by regulating the production of the extracellular matrix and coordinating the balance of signals inside and outside the cell. The authors present the structure of AP-1/Fra-2 [157], its expression, the importance of Fra-2 in tissue development, and its regulation. They then review the role of Fra-2 in the development of respiratory diseases, chronic obstructive pulmonary disease, pulmonary fibrosis, asthma, and non-small cell lung cancer.

## Figures and Tables

**Figure 1:**
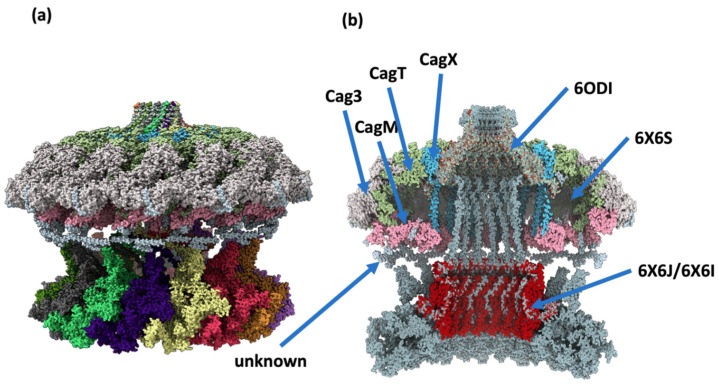
Assembly of the CagY protein with other protein structures of the T4SS from cryo-EM studies. (**a**) The model of CagY fitted with the structure of the T4SS from cryo-EM (PDB ID 6 × 6S [59]). (**b**) A transversal section of the assembly, showing that the most internal structure corresponds to the CagY multimer (in light grey). Other proteins of the T4SS are displayed in different colours. The structure marked as “unknown” corresponds to a non-identified protein, which is in close proximity to the protuberances of the long middle repeat region of the predicted model of CagY (taken from [48]).

**Figure 2:**
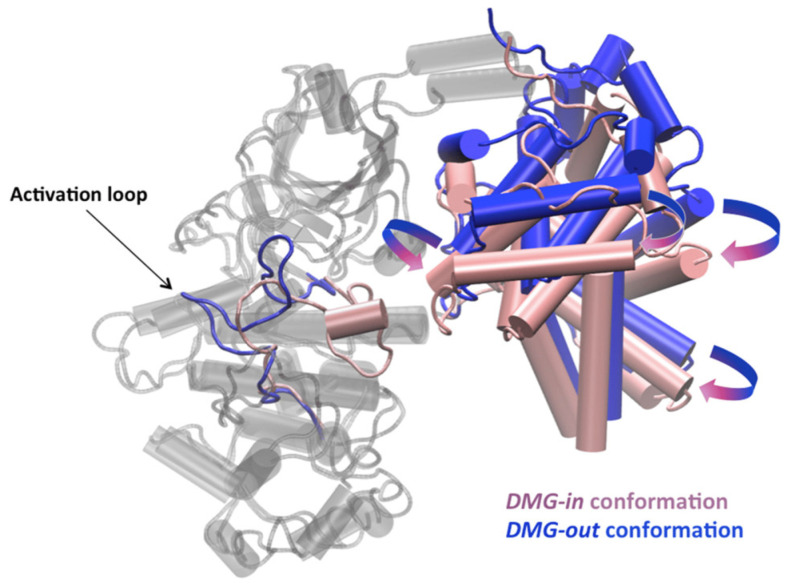
DMG-in and DMG-out conformations of the CDK8–CycC complexes. CDK8 structures are coloured in grey, except the activation loop. The activation loop and the CycC structures are coloured in pink in the DMG-in conformation and in blue in the DMG-out conformation (taken from [60]). DMG (Asp-Met-Gly) motif ranges from residues 173 to 175.

**Figure 3:**
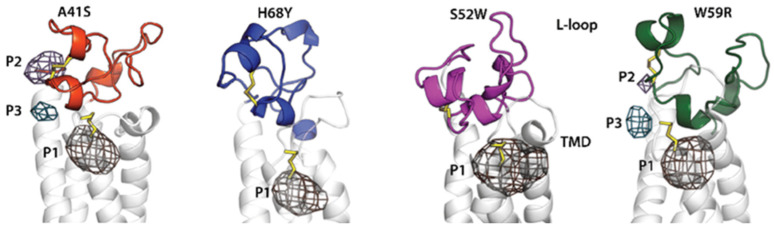
Pockets in hVKORC1 mutants. Pockets localised in four mutants. Protein is shown as a cartoon with the L-loop distinguished by color: hVKORC1^A41S^ in orange-red; hVKORC1^H68Y^ in dark blue; hVKORC1^S52W^ in fuchsia; and hVKORC1^W59R^ in dark green. Disulphide bonds and crucial residues are in sticks; pockets are delimited by meshed contours (adapted from [65]).

**Figure 4:**
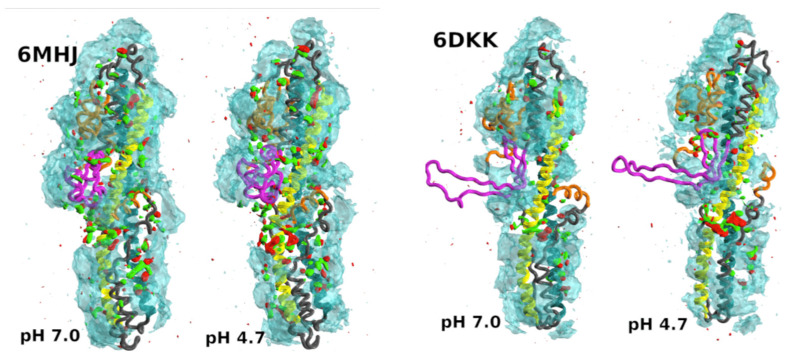
Isosurfaces of the spatial density function of water and ethanol atoms around the protein. Cyan isosurface: water oxygen atoms; red and green: oxygen and methyl carbon atoms, respectively. The isosurfaces are represented at the same isodensity level (0.0115) for both water and ethanol. Data were collected from the three replicates of the trajectory. The proteins are PDB entries: six MHJ determined at a pH 8.5 and six DKK were determined at a pH 5.1 [81]. The helices 1 (cyan) and 2 (yellow) are shown for the two PDB structures. Green spheres represent the hydrophobic residues in these helices. The switch is coloured in magenta, and the N-terminal domain and the C-terminal *α* helix in orange (taken from [70]).

**Figure 5:**
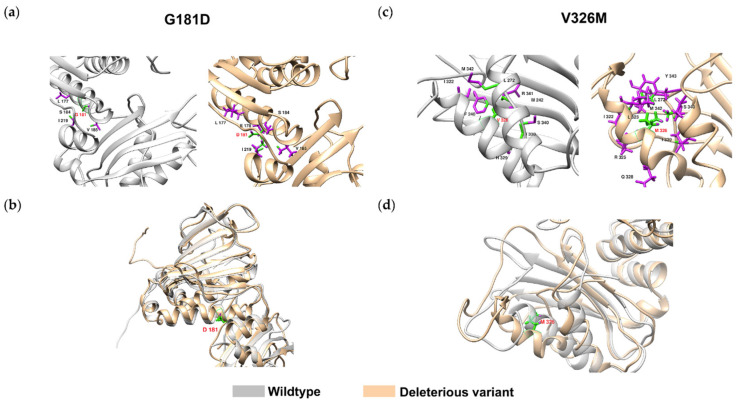
Structural change in MLH1 by G181D and V326M. (**a**) The wild-type G181 interacted with four residues (L177, S184, V185, and I219), whereas the variant D181 interacted with five residues (L177, E178, S184, V185, and I219). (**b**) The D181 caused instability of αG and further affected the MutS-HI domain. (**c**) The wild-typeV326 interacted with nine residues (F240, M242, L272, I322, N329, I330, S340, R341, and M342), and the M326 interacted with altered nine residues (L272, I322, L323, R325, Q328, I330, S340, M342, and Y343). (**d**) The M326 did not interact with the β sheet and caused the αI helix to detach. Grey: wild-type; peach: variant; green: interacting atoms; purple: non-interacting atoms; red label: wild-type and variants; black label: interacting residues (taken from [82]).

**Figure 6:**
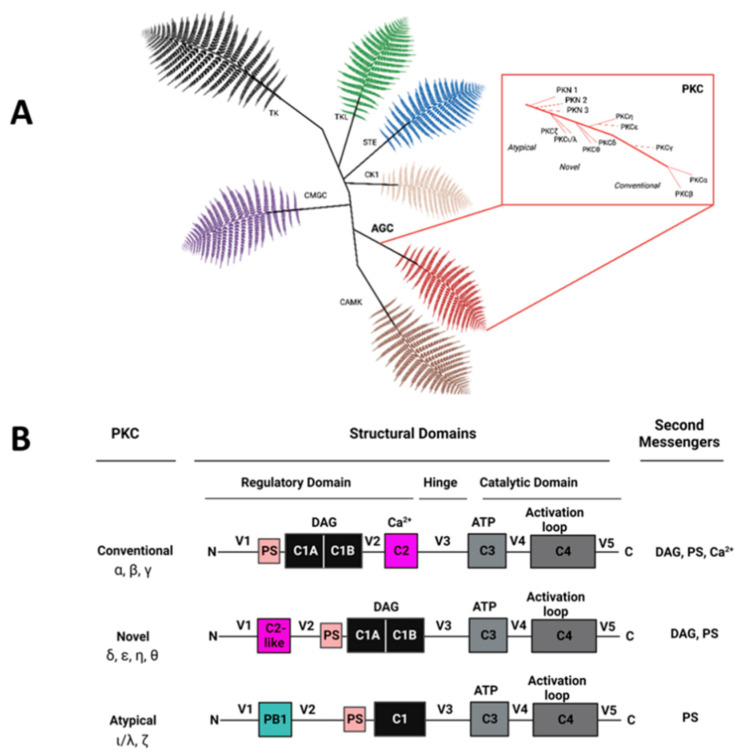
PKC isozyme diversity. (**A**) The human kinomephylogeny is extensive, and the PKC family is one member of the AGC superfamily (bottom right), with three groups of conventional, novel, and atypical PKC isozymes. (**B**) PKC isozymes are homologous but contain a distinct set of structural domains responsible for their diverse functions and interactions with second messengers and other binding partners. All PKC family members are constituted by four conserved domains (C1–C4) separated by a hinge region. The pseudo-substrate site (PS) keeps the protein in its inactive form. Second messengers are indicated on the right side of the picture (taken from [101]).

**Figure 7:**
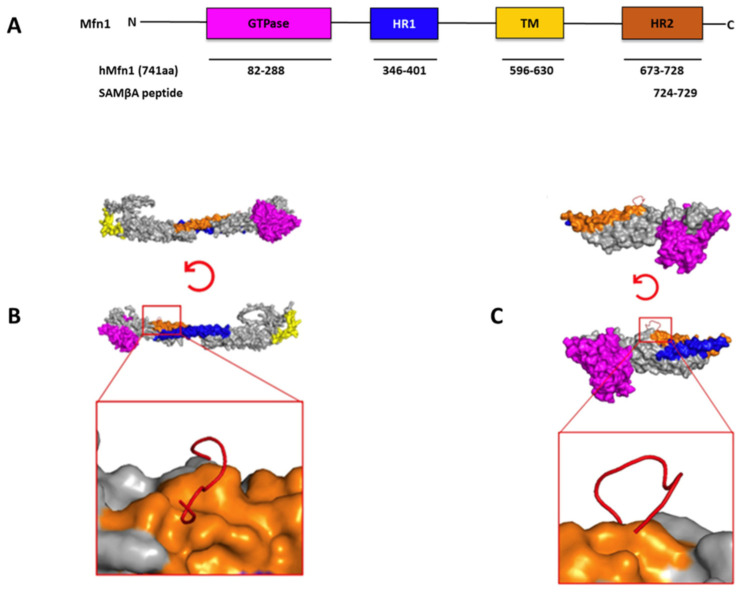
Structural domains of Mfn1 and peptides derived from substrate protein–protein interaction sites. A sequence corresponding to the homologous domain between PKCβII (P05771-2; residues 625–629) and Mfn1 (26251799; residues 724–729) was identified, and the SAMβA peptide corresponding to this sequence was developed. (**A**) The structural and functional domains of full-length mitofusin-1 (Mfn1) (741 AAs). The GTPase domain is shown in magenta, heptad repeat (HR1) coiled-coil regions 1 in blue, the transmembrane (TM) domain is shown in yellow, and heptad repeat (HR2) coiled-coil regions 2 in orange. (**B**) Molecular docking results for the interaction of SAMβA (RNAENFDRF) and Mfn1 to the available crystal structure (PDB: 5GO4 [118]) or (**C**) AlphaFold predicted model (Q8IWA4). To help stabilise the α-helix structure, amino acids were added at the C- and N-terminus of the peptide. Mfn1 is shown by cartoon representation coloured in (GTPase—magenta; HR1—blue; HR2—orange; and TM—yellow) and the peptide is shown in red cartoon structure. Based on the docking analysis, the peptide is docked to the HR2, which is source domain for its rational design (taken from [134]).

## References

[1] Baker D., Sali A. (2001). Protein structure prediction and structural genomics. Science.

[2] Lee C., Su B.H., Tseng Y.J. (2022). Comparative studies of alphafold, rosettafold and modeller: A case study involving the use of g-protein-coupled receptors. Brief. Bioinform..

[3] Webb B., Sali A. (2016). Comparative protein structure modeling using modeller. Curr. Protoc. Bioinform..

[4] Sali A., Blundell T.L. (1993). Comparative protein modelling by satisfaction of spatial restraints. J. Mol. Biol..

[5] Kelley L.A., Mezulis S., Yates C.M., Wass M.N., Sternberg M.J. (2015). The phyre2 web portal for protein modeling, prediction and analysis. Nat. Protoc..

[6] Yang J., Zhang Y. (2015). I-tasser server: New development for protein structure and function predictions. Nucleic Acids Res..

[7] Zhou X., Zheng W., Li Y., Pearce R., Zhang C., Bell E.W., Zhang G., Zhang Y. (2022). I-tasser-mtd: A deep-learning-based platform for multi-domain protein structure and function prediction. Nat. Protoc..

[8] Alford R.F., Leaver-Fay A., Jeliazkov J.R., O’Meara M.J., DiMaio F.P., Park H., Shapovalov M.V., Renfrew P.D., Mulligan V.K., Kappel K. (2017). The rosetta all-atom energy function for macromolecular modeling and design. J. Chem. Theory Comput..

[9] Dauparas J., Anishchenko I., Bennett N., Bai H., Ragotte R.J., Milles L.F., Wicky B.I.M., Courbet A., de Haas R.J., Bethel N. (2022). Robust deep learning-based protein sequence design using proteinmpnn. Science.

[10] Jumper J., Evans R., Pritzel A., Green T., Figurnov M., Ronneberger O., Tunyasuvunakool K., Bates R., Žídek A., Potapenko A. (2021). Highly accurate protein structure prediction with alphafold. Nature.

[11] Abramson J., Adler J., Dunger J., Evans R., Green T., Pritzel A., Ronneberger O., Willmore L., Ballard A.J., Bambrick J. (2024). Accurate structure prediction of biomolecular interactions with alphafold 3. Nature.

[12] Manfredi M., Savojardo C., Iardukhin G., Salomoni D., Costantini A., Martelli P.L., Casadio R. (2024). Alpha&esmhfolds: A web server for comparing alphafold2 and esmfold models of the human reference proteome. J. Mol. Biol..

[13] Lin Z., Akin H., Rao R., Hie B., Zhu Z., Lu W., Smetanin N., Verkuil R., Kabeli O., Shmueli Y. (2023). Evolutionary-scale prediction of atomic-level protein structure with a language model. Science.

[14] Tourlet S., Radjasandirane R., Diharce J., de Brevern A.G. (2023). Alphafold2 update and perspectives. BioMedInformatics.

[15] Radjasandirane R., de Brevern A.G. (2024). Alphafold2 for protein structure prediction: Best practices and critical analyses. Methods Mol. Biol..

[16] Varadi M., Anyango S., Deshpande M., Nair S., Natassia C., Yordanova G., Yuan D., Stroe O., Wood G., Laydon A. (2022). Alphafold protein structure database: Massively expanding the structural coverage of protein-sequence space with high-accuracy models. Nucleic Acids Res..

[17] Tunyasuvunakool K., Adler J., Wu Z., Green T., Zielinski M., Žídek A., Bridgland A., Cowie A., Meyer C., Laydon A. (2021). Highly accurate protein structure prediction for the human proteome. Nature.

[18] de Brevern A.G. (2023). An agnostic analysis of the human alphafold2 proteome using local protein conformations. Biochimie.

[19] Niazi S.K., Mariam Z., Paracha R.Z. (2024). Limitations of protein structure prediction algorithms in therapeutic protein development. BioMedInformatics.

[20] Ma W., Zhang S., Li Z., Jiang M., Wang S., Lu W., Bi X., Jiang H., Zhang H., Wei Z. (2022). Enhancing protein function prediction performance by utilizing alphafold-predicted protein structures. J. Chem. Inf. Model..

[21] Whisstock J.C., Lesk A.M. (2003). Prediction of protein function from protein sequence and structure. Q. Rev. Biophys..

[22] Bitard-Feildel T., Lamiable A., Mornon J.P., Callebaut I. (2018). Order in disorder as observed by the “hydrophobic cluster analysis” of protein sequences. Proteomics.

[23] DeForte S., Uversky V.N. (2016). Order, disorder, and everything in between. Molecules.

[24] Huang A., Stultz C.M. (2009). Finding order within disorder: Elucidating the structure of proteins associated with neurodegenerative disease. Future Med. Chem..

[25] de Brevern A.G. (2020). Analysis of protein disorder predictions in the light of a protein structural alphabet. Biomolecules.

[26] Orellana L. (2019). Large-scale conformational changes and protein function: Breaking the in silico barrier. Front. Mol. Biosci..

[27] Ziarek J.J., Baptista D., Wagner G. (2018). Recent developments in solution nuclear magnetic resonance (nmr)-based molecular biology. J. Mol. Med..

[28] Bowman G.R. (2024). Alphafold and protein folding: Not dead yet! The frontier is conformational ensembles. Annu. Rev. Biomed. Data Sci..

[29] Gianni S., Jemth P. (2023). Allostery frustrates the experimentalist. J. Mol. Biol..

[30] Ray D., Parrinello M. (2023). Kinetics from metadynamics: Principles, applications, and outlook. J. Chem. Theory Comput..

[31] Van Der Spoel D., Lindahl E., Hess B., Groenhof G., Mark A.E., Berendsen H.J. (2005). Gromacs: Fast, flexible, and free. J. Comput. Chem..

[32] Brooks B.R., Brooks C.L., Mackerell A.D., Nilsson L., Petrella R.J., Roux B., Won Y., Archontis G., Bartels C., Boresch S. (2009). Charmm: The biomolecular simulation program. J. Comput. Chem..

[33] Collier T.A., Piggot T.J., Allison J.R. (2020). Molecular dynamics simulation of proteins. Methods Mol. Biol..

[34] Filipe H.A.L., Loura L.M.S. (2022). Molecular dynamics simulations: Advances and applications. Molecules.

[35] Nam K.H. (2022). Molecular dynamics-from macromolecule to small molecules. Int. J. Mol. Sci..

[36] Wu X., Xu L.Y., Li E.M., Dong G. (2022). Application of molecular dynamics simulation in biomedicine. Chem. Biol. Drug Des..

[37] Anies S., Jallu V., Diharce J., Narwani T.J., de Brevern A.G. (2022). Analysis of integrin α(iib) subunit dynamics reveals long-range effects of missense mutations on calf domains. Int. J. Mol. Sci..

[38] Goguet M., Narwani T.J., Petermann R., Jallu V., de Brevern A.G. (2017). In silico analysis of glanzmann variants of calf-1 domain of α(iib)β(3) integrin revealed dynamic allosteric effect. Sci. Rep..

[39] Sang P., Hu W., Ye Y.J., Li L.H., Zhang C., Xie Y.H., Meng Z.H. (2017). In silico screening, molecular docking, and molecular dynamics studies of snp-derived human p5cr mutants. J. Biomol. Struct. Dyn..

[40] Sneha P., Doss C.G. (2016). Molecular dynamics: New frontier in personalized medicine. Adv. Protein Chem. Struct. Biol..

[41] Elangeeb M.E., Elfaki I., Eleragi A.M.S., Ahmed E.M., Mir R., Alzahrani S.M., Bedaiwi R.I., Alharbi Z.M., Mir M.M., Ajmal M.R. (2024). Molecular dynamics simulation of kir6.2 variants reveals potential association with diabetes mellitus. Molecules.

[42] Cortina G.A., Kasson P.M. (2018). Predicting allostery and microbial drug resistance with molecular simulations. Curr. Opin. Struct. Biol..

[43] Guo J., Zhou H.X. (2016). Protein allostery and conformational dynamics. Chem. Rev..

[44] Hertig S., Latorraca N.R., Dror R.O. (2016). Revealing atomic-level mechanisms of protein allostery with molecular dynamics simulations. PLoS Comput. Biol..

[45] Wodak S.J., Paci E., Dokholyan N.V., Berezovsky I.N., Horovitz A., Li J., Hilser V.J., Bahar I., Karanicolas J., Stock G. (2019). Allostery in its many disguises: From theory to applications. Structure.

[46] Xiong L., Liu Z. (2015). Molecular dynamics study on folding and allostery in rfah. Proteins.

[47] Allain A., Chauvot de Beauchêne I., Langenfeld F., Guarracino Y., Laine E., Tchertanov L. (2014). Allosteric pathway identification through network analysis: From molecular dynamics simulations to interactive 2d and 3d graphs. Faraday Discuss..

[48] López-Luis M.A., Soriano-Pérez E.E., Parada-Fabián J.C., Torres J., Maldonado-Rodríguez R., Méndez-Tenorio A. (2023). A proposal for a consolidated structural model of the cagy protein of helicobacter pylori. Int. J. Mol. Sci..

[49] Camilo V., Sugiyama T., Touati E. (2017). Pathogenesis of helicobacter pylori infection. Helicobacter.

[50] Malfertheiner P., Camargo M.C., El-Omar E., Liou J.M., Peek R., Schulz C., Smith S.I., Suerbaum S. (2023). Helicobacter pylori infection. Nat. Rev. Dis. Primers.

[51] Salvatori S., Marafini I., Laudisi F., Monteleone G., Stolfi C. (2023). Helicobacter pylori and gastric cancer: Pathogenetic mechanisms. Int. J. Mol. Sci..

[52] Sun Q., Yuan C., Zhou S., Lu J., Zeng M., Cai X., Song H. (2023). Helicobacter pylori infection: A dynamic process from diagnosis to treatment. Front. Cell. Infect. Microbiol..

[53] Odenbreit S., Püls J., Sedlmaier B., Gerland E., Fischer W., Haas R. (2000). Translocation of helicobacter pylori caga into gastric epithelial cells by type iv secretion. Science.

[54] Akopyants N.S., Clifton S.W., Kersulyte D., Crabtree J.E., Youree B.E., Reece C.A., Bukanov N.O., Drazek E.S., Roe B.A., Berg D.E. (1998). Analyses of the cag pathogenicity island of helicobacter pylori. Mol. Microbiol..

[55] Mirdita M., Schütze K., Moriwaki Y., Heo L., Ovchinnikov S., Steinegger M. (2022). Colabfold: Making protein folding accessible to all. Nat. Methods.

[56] Wiederstein M., Sippl M.J. (2007). Prosa-web: Interactive web service for the recognition of errors in three-dimensional structures of proteins. Nucleic Acids Res..

[57] Colovos C., Yeates T.O. (1993). Verification of protein structures: Patterns of nonbonded atomic interactions. Protein Sci. Publ. Protein Soc..

[58] Studer G., Rempfer C., Waterhouse A.M., Gumienny R., Haas J., Schwede T. (2020). Qmeandisco-distance constraints applied on model quality estimation. Bioinformatics.

[59] Sheedlo M.J., Chung J.M., Sawhney N., Durie C.L., Cover T.L., Ohi M.D., Lacy D.B. (2020). Cryo-em reveals species-specific components within the helicobacter pylori cag type iv secretion system core complex. eLife.

[60] Ziada S., Diharce J., Serillon D., Bonnet P., Aci-Sèche S. (2024). Highlighting the major role of cyclin c in cyclin-dependent kinase 8 activity through molecular dynamics simulations. Int. J. Mol. Sci..

[61] Zabihi M., Lotfi R., Yousefi A.M., Bashash D. (2023). Cyclins and cyclin-dependent kinases: From biology to tumorigenesis and therapeutic opportunities. J. Cancer Res. Clin. Oncol..

[62] Rzymski T., Mikula M., Wiklik K., Brzózka K. (2015). Cdk8 kinase—An emerging target in targeted cancer therapy. Biochim. Biophys. Acta.

[63] Philip S., Kumarasiri M., Teo T., Yu M., Wang S. (2018). Cyclin-dependent kinase 8: A new hope in targeted cancer therapy?. J. Med. Chem..

[64] Zehra, Hussain A., AlAjmi M.F., Ishrat R., Hassan M.I. (2024). Enriching anticancer drug pipeline with potential inhibitors of cyclin-dependent kinase-8 identified from natural products. Omics J. Integr. Biol..

[65] Botnari M., Tchertanov L. (2024). Synergy of mutation-induced effects in human vitamin k epoxide reductase: Perspectives and challenges for allo-network modulator design. Int. J. Mol. Sci..

[66] Ledoux J., Stolyarchuk M., Bachelier E., Trouvé A., Tchertanov L. (2022). Human vitamin k epoxide reductase as a target of its redox protein. Int. J. Mol. Sci..

[67] Stolyarchuk M., Botnari M., Tchertanov L. (2024). Vitamin k epoxide reductase complex-protein disulphide isomerase assemblies in the thiol-disulphide exchange reactions: Portrayal of precursor-to-successor complexes. Int. J. Mol. Sci..

[68] Stolyarchuk M., Ledoux J., Maignant E., Trouvé A., Tchertanov L. (2021). Identification of the primary factors determining thespecificity of human vkorc1 recognition by thioredoxin-fold proteins. Int. J. Mol. Sci..

[69] Chatron N., Chalmond B., Trouvé A., Benoît E., Caruel H., Lattard V., Tchertanov L. (2017). Identification of the functional states of human vitamin k epoxide reductase from molecular dynamics simulations. RSC Adv..

[70] Delort A., Cottone G., Malliavin T.E., Müller M.M. (2024). Conformational space of the translocation domain of botulinum toxin: Atomistic modeling and mesoscopic description of the coiled-coil helix bundle. Int. J. Mol. Sci..

[71] Dong M., Masuyer G., Stenmark P. (2019). Botulinum and tetanus neurotoxins. Annu. Rev. Biochem..

[72] Coetzee S., Nunez N., Belaunzaran M., Mark J., Stickler M.A. (2023). Beyond wrinkles: A comprehensive review of the uses of botulinum toxin. J. Drugs Dermatol..

[73] Lacy D.B., Tepp W., Cohen A.C., DasGupta B.R., Stevens R.C. (1998). Crystal structure of botulinum neurotoxin type a and implications for toxicity. Nat. Struct. Biol..

[74] Kumaran D., Eswaramoorthy S., Furey W., Navaza J., Sax M., Swaminathan S. (2009). Domain organization in clostridium botulinum neurotoxin type e is unique: Its implication in faster translocation. J. Mol. Biol..

[75] Cottone G., Chiodo L., Maragliano L., Popoff M.R., Rasetti-Escargueil C., Lemichez E., Malliavin T.E. (2022). In silico conformational features of botulinum toxins a1 and e1 according to intraluminal acidification. Toxins.

[76] Fierling J., Johner A., Kulić I.M., Mohrbach H., Müller M.M. (2016). How bio-filaments twist membranes. Soft Matter.

[77] Ramirez-Diaz D.A., Merino-Salomón A., Meyer F., Heymann M., Rivas G., Bramkamp M., Schwille P. (2021). Ftsz induces membrane deformations via torsional stress upon gtp hydrolysis. Nat. Commun..

[78] Chiaruttini N., Redondo-Morata L., Colom A., Humbert F., Lenz M., Scheuring S., Roux A. (2015). Relaxation of loaded escrt-iii spiral springs drives membrane deformation. Cell.

[79] Moser von Filseck J., Barberi L., Talledge N., Johnson I.E., Frost A., Lenz M., Roux A. (2020). Anisotropic escrt-iii architecture governs helical membrane tube formation. Nat. Commun..

[80] Pannuzzo M., McDargh Z.A., Deserno M. (2018). The role of scaffold reshaping and disassembly in dynamin driven membrane fission. eLife.

[81] Lam K.H., Guo Z., Krez N., Matsui T., Perry K., Weisemann J., Rummel A., Bowen M.E., Jin R. (2018). A viral-fusion-peptide-like molecular switch drives membrane insertion of botulinum neurotoxin a1. Nat. Commun..

[82] Tam B., Qin Z., Zhao B., Sinha S., Lei C.L., Wang S.M. (2024). Classification of mlh1 missense vus using protein structure-based deep learning-ramachandran plot-molecular dynamics simulations method. Int. J. Mol. Sci..

[83] Radjasandirane R., Diharce J., Gelly J.-C., de Brevern A.G. (2024). Assessment of variant effect predictors unveils variants difficulty as a critical performance indicator. bioRxiv.

[84] Ng P.C., Henikoff S. (2003). Sift: Predicting amino acid changes that affect protein function. Nucleic Acids Res..

[85] Adzhubei I.A., Schmidt S., Peshkin L., Ramensky V.E., Gerasimova A., Bork P., Kondrashov A.S., Sunyaev S.R. (2010). A method and server for predicting damaging missense mutations. Nat. Methods.

[86] Landrum M.J., Lee J.M., Riley G.R., Jang W., Rubinstein W.S., Church D.M., Maglott D.R. (2014). Clinvar: Public archive of relationships among sequence variation and human phenotype. Nucleic Acids Res..

[87] Richards S., Aziz N., Bale S., Bick D., Das S., Gastier-Foster J., Grody W.W., Hegde M., Lyon E., Spector E. (2015). Standards and guidelines for the interpretation of sequence variants: A joint consensus recommendation of the american college of medical genetics and genomics and the association for molecular pathology. Genet. Med. Off. J. Am. Coll. Med. Genet..

[88] Cheng J., Novati G., Pan J., Bycroft C., Žemgulytė A., Applebaum T., Pritzel A., Wong L.H., Zielinski M., Sargeant T. (2023). Accurate proteome-wide missense variant effect prediction with alphamissense. Science.

[89] Li C., Zhi D., Wang K., Liu X. (2022). Metarnn: Differentiating rare pathogenic and rare benign missense snvs and indels using deep learning. Genome Med..

[90] Jia P., Chai W. (2018). The mlh1 atpase domain is needed for suppressing aberrant formation of interstitial telomeric sequences. DNA Repair..

[91] Ryan N.A.J., Glaire M.A., Blake D., Cabrera-Dandy M., Evans D.G., Crosbie E.J. (2019). The proportion of endometrial cancers associated with lynch syndrome: A systematic review of the literature and meta-analysis. Genet. Med. Off. J. Am. Coll. Med. Genet..

[92] Tam B., Sinha S., Wang S.M. (2020). Combining ramachandran plot and molecular dynamics simulation for structural-based variant classification: Using tp53 variants as model. Comput. Struct. Biotechnol. J..

[93] Park S.W., Lee B.H., Song S.H., Kim M.K. (2023). Revisiting the ramachandran plot based on statistical analysis of static and dynamic characteristics of protein structures. J. Struct. Biol..

[94] Ramachandran G.N., Ramakrishnan C., Sasisekharan V. (1963). Stereochemistry of polypeptide chain configurations. J. Mol. Biol..

[95] Ravikumar A., Ramakrishnan C., Srinivasan N. (2019). Stereochemical assessment of (φ,ψ) outliers in protein structures using bond geometry-specific ramachandran steric-maps. Structure.

[96] Lakshmi B., Ramakrishnan C., Archunan G., Sowdhamini R., Srinivasan N. (2014). Investigations of ramachandran disallowed conformations in protein domain families. Int. J. Biol. Macromol..

[97] Carugo O., Djinovic-Carugo K. (2013). Half a century of ramachandran plots. Acta Crystallogr. Sect. D Biol. Crystallogr..

[98] Carugo O., Djinović-Carugo K. (2013). A proteomic ramachandran plot (prplot). Amino Acids.

[99] Wu H., Zeng H., Lam R., Tempel W., Kerr I.D., Min J. (2015). Structure of the human mlh1 n-terminus: Implications for predisposition to lynch syndrome. Acta Crystallogr. Sect. F Struct. Biol. Commun..

[100] Shen M.Y., Sali A. (2006). Statistical potential for assessment and prediction of protein structures. Protein Sci. Publ. Protein Soc..

[101] Silnitsky S., Rubin S.J.S., Zerihun M., Qvit N. (2023). An update on protein kinases as therapeutic targets-part i: Protein kinase c activation and its role in cancer and cardiovascular diseases. Int. J. Mol. Sci..

[102] Johnson J.L., Yaron T.M., Huntsman E.M., Kerelsky A., Song J., Regev A., Lin T.Y., Liberatore K., Cizin D.M., Cohen B.M. (2023). An atlas of substrate specificities for the human serine/threonine kinome. Nature.

[103] Benn C.L., Dawson L.A. (2020). Clinically precedented protein kinases: Rationale for their use in neurodegenerative disease. Front. Aging Neurosci..

[104] Krebs E.G., Fischer E.H. (1956). The phosphorylase b to a converting enzyme of rabbit skeletal muscle. Biochim. Biophys. Acta.

[105] Cohen P. (2002). Protein kinases—The major drug targets of the twenty-first century?. Nat. Rev. Drug Discov..

[106] Attwood M.M., Fabbro D., Sokolov A.V., Knapp S., Schiöth H.B. (2021). Trends in kinase drug discovery: Targets, indications and inhibitor design. Nat. Rev. Drug Discov..

[107] Levin D.E., Fields F.O., Kunisawa R., Bishop J.M., Thorner J. (1990). A candidate protein kinase c gene, pkc1, is required for the s. Cerevisiae cell cycle. Cell.

[108] Watanabe M., Chen C.Y., Levin D.E. (1994). Saccharomyces cerevisiae pkc1 encodes a protein kinase c (pkc) homolog with a substrate specificity similar to that of mammalian pkc. J. Biol. Chem..

[109] Gould C.M., Newton A.C. (2008). The life and death of protein kinase c. Curr. Drug Targets.

[110] Violin J.D., Zhang J., Tsien R.Y., Newton A.C. (2003). A genetically encoded fluorescent reporter reveals oscillatory phosphorylation by protein kinase c. J. Cell Biol..

[111] Bartlett P.J., Young K.W., Nahorski S.R., Challiss R.A. (2005). Single cell analysis and temporal profiling of agonist-mediated inositol 1,4,5-trisphosphate, ca2+, diacylglycerol, and protein kinase c signaling using fluorescent biosensors. J. Biol. Chem..

[112] Langeberg L.K., Scott J.D. (2015). Signalling scaffolds and local organization of cellular behaviour. Nat. Rev. Mol. Cell Biol..

[113] Finger E.C., Castellini L., Rankin E.B., Vilalta M., Krieg A.J., Jiang D., Banh A., Zundel W., Powell M.B., Giaccia A.J. (2015). Hypoxic induction of akap12 variant 2 shifts pka-mediated protein phosphorylation to enhance migration and metastasis of melanoma cells. Proc. Natl. Acad. Sci. USA.

[114] Obsilova V., Obsil T. (2020). The 14-3-3 proteins as important allosteric regulators of protein kinases. Int. J. Mol. Sci..

[115] Zhang J., Yang P.L., Gray N.S. (2009). Targeting cancer with small molecule kinase inhibitors. Nat. Rev. Cancer.

[116] Bhullar K.S., Lagarón N.O., McGowan E.M., Parmar I., Jha A., Hubbard B.P., Rupasinghe H.P.V. (2018). Kinase-targeted cancer therapies: Progress, challenges and future directions. Mol. Cancer.

[117] Hu J.X., Zhao C.F., Chen W.B., Liu Q.C., Li Q.W., Lin Y.Y., Gao F. (2021). Pancreatic cancer: A review of epidemiology, trend, and risk factors. World J. Gastroenterol..

[118] Kawano T., Tachibana Y., Inokuchi J., Kang J.H., Murata M., Eto M. (2021). Identification of activated protein kinase cα (pkcα) in the urine of orthotopic bladder cancer xenograft model as a potential biomarker for the diagnosis of bladder cancer. Int. J. Mol. Sci..

[119] Chou W.H., Messing R.O. (2005). Protein kinase c isozymes in stroke. Trends Cardiovasc. Med..

[120] Fuller S.J., Osborne S.A., Leonard S.J., Hardyman M.A., Vaniotis G., Allen B.G., Sugden P.H., Clerk A. (2015). Cardiac protein kinases: The cardiomyocyte kinome and differential kinase expression in human failing hearts. Cardiovasc. Res..

[121] Virani S.S., Alonso A., Aparicio H.J., Benjamin E.J., Bittencourt M.S., Callaway C.W., Carson A.P., Chamberlain A.M., Cheng S., Delling F.N. (2021). Heart disease and stroke statistics-2021 update: A report from the american heart association. Circulation.

[122] Miao L.N., Pan D., Shi J., Du J.P., Chen P.F., Gao J., Yu Y., Shi D.Z., Guo M. (2022). Role and mechanism of pkc-δ for cardiovascular disease: Current status and perspective. Front. Cardiovasc. Med..

[123] Soares A.C., Fonseca D.A. (2020). Cardiovascular diseases: A therapeutic perspective around the clock. Drug Discov. Today.

[124] Schwegmann A., Guler R., Cutler A.J., Arendse B., Horsnell W.G., Flemming A., Kottmann A.H., Ryan G., Hide W., Leitges M. (2007). Protein kinase c delta is essential for optimal macrophage-mediated phagosomal containment of listeria monocytogenes. Proc. Natl. Acad. Sci. USA.

[125] Mondrinos M.J., Kennedy P.A., Lyons M., Deutschman C.S., Kilpatrick L.E. (2013). Protein kinase c and acute respiratory distress syndrome. Shock.

[126] Gauron M.C., Newton A.C., Colombo M.I. (2021). Pkcα is recruited to staphylococcus aureus-containing phagosomes and impairs bacterial replication by inhibition of autophagy. Front. Immunol..

[127] Kim P.M., Kornberg M.D. (2022). Targeting pkc in microglia to promote remyelination and repair in the cns. Curr. Opin. Pharmacol..

[128] Lee K.W., Kim S.G., Kim H.P., Kwon E., You J., Choi H.J., Park J.H., Kang B.C., Im S.A., Kim T.Y. (2008). Enzastaurin, a protein kinase c beta inhibitor, suppresses signaling through the ribosomal s6 kinase and bad pathways and induces apoptosis in human gastric cancer cells. Cancer Res..

[129] Wu-Zhang A.X., Newton A.C. (2013). Protein kinase c pharmacology: Refining the toolbox. Biochem. J..

[130] Raghuvanshi R., Bharate S.B. (2020). Preclinical and clinical studies on bryostatins, a class of marine-derived protein kinase c modulators: A mini-review. Curr. Top. Med. Chem..

[131] Rahimova N., Cooke M., Zhang S., Baker M.J., Kazanietz M.G. (2020). The pkc universe keeps expanding: From cancer initiation to metastasis. Adv. Biol. Regul..

[132] Kawano T., Inokuchi J., Eto M., Murata M., Kang J.H. (2021). Activators and inhibitors of protein kinase c (pkc): Their applications in clinical trials. Pharmaceutics.

[133] Ghoreschi K., Laurence A., O’Shea J.J. (2009). Selectivity and therapeutic inhibition of kinases: To be or not to be?. Nat. Immunol..

[134] Zerihun M., Rubin S.J.S., Silnitsky S., Qvit N. (2023). An update on protein kinases as therapeutic targets-part ii: Peptides as allosteric protein kinase c modulators targeting protein-protein interactions. Int. J. Mol. Sci..

[135] de la Torre B.G., Albericio F. (2024). The pharmaceutical industry in 2023: An analysis of fda drug approvals from the perspective of molecules. Molecules.

[136] Cohen P., Cross D., Jänne P.A. (2021). Kinase drug discovery 20 years after imatinib: Progress and future directions. Nat. Rev. Drug Discov..

[137] Mobitz H., Jahnke W., Cowan-Jacob S.W. (2017). Expanding the opportunities for modulating kinase targets with allosteric approaches. Curr. Top. Med. Chem..

[138] Palmieri L., Rastelli G. (2013). Ac helix displacement as a general approach for allosteric modulation of protein kinases. Drug Discov. Today.

[139] Zorba A., Nguyen V., Koide A., Hoemberger M., Zheng Y., Kutter S., Kim C., Koide S., Kern D. (2019). Allosteric modulation of a human protein kinase with monobodies. Proc. Natl. Acad. Sci. USA.

[140] Ivanov A.A., Khuri F.R., Fu H. (2013). Targeting protein-protein interactions as an anticancer strategy. Trends Pharmacol. Sci..

[141] Rubin S.J.S., Tal-Gan Y., Gilon C., Qvit N. (2018). Conversion of protein active regions into peptidomimetic therapeutic leads using backbone cyclization and cycloscan—How to do it yourself!. Curr. Top. Med. Chem..

[142] Qvit N., Kornfeld O.S., Mochly-Rosen D. (2016). Engineered substrate-specific delta pkc antagonists to enhance cardiac therapeutics. Angew. Chem..

[143] Qvit N., Mochly-Rosen D. (2010). Highly specific modulators of protein kinase c localization: Applications to heart failure. Drug Discov. Today. Dis. Mech..

[144] Qvit N., Mochly-Rosen D. (2014). The many hats of protein kinase cδ: One enzyme with many functions. Biochem. Soc. Trans..

[145] House C., Kemp B.E. (1987). Protein kinase c contains a pseudosubstrate prototope in its regulatory domain. Science.

[146] Makowske M., Rosen O.M. (1989). Complete activation of protein kinase c by an antipeptide antibody directed against the pseudosubstrate prototope. J. Biol. Chem..

[147] Jayaram D.R., Frost S., Argov C., Liju V.B., Anto N.P., Muraleedharan A., Ben-Ari A., Sinay R., Smoly I., Novoplansky O. (2021). Unraveling the hidden role of a uorf-encoded peptide as a kinase inhibitor of pkcs. Proc. Natl. Acad. Sci. USA.

[148] Ubersax J.A., Ferrell J.E. (2007). Mechanisms of specificity in protein phosphorylation. Nat. Rev. Mol. Cell Biol..

[149] Ferreira J.C.B., Campos J.C., Qvit N., Qi X., Bozi L.H.M., Bechara L.R.G., Lima V.M., Queliconi B.B., Disatnik M.H., Dourado P.M.M. (2019). A selective inhibitor of mitofusin 1-βiipkc association improves heart failure outcome in rats. Nat. Commun..

[150] Pawson T., Scott J.D. (1997). Signaling through scaffold, anchoring, and adaptor proteins. Science.

[151] Hakes L., Lovell S.C., Oliver S.G., Robertson D.L. (2007). Specificity in protein interactions and its relationship with sequence diversity and coevolution. Proc. Natl. Acad. Sci. USA.

[152] Nguyen T.A., Takemoto L.J., Takemoto D.J. (2004). Inhibition of gap junction activity through the release of the c1b domain of protein kinase cgamma (pkcgamma) from 14-3-3: Identification of pkcgamma-binding sites. J. Biol. Chem..

[153] Liron T., Chen L.E., Khaner H., Vallentin A., Mochly-Rosen D. (2007). Rational design of a selective antagonist of epsilon protein kinase c derived from the selective allosteric agonist, pseudo-rack peptide. J. Mol. Cell. Cardiol..

[154] Clark J.D., Lin L.L., Kriz R.W., Ramesha C.S., Sultzman L.A., Lin A.Y., Milona N., Knopf J.L. (1991). A novel arachidonic acid-selective cytosolic pla2 contains a ca(2+)-dependent translocation domain with homology to pkc and gap. Cell.

[155] Dunn J., McCuaig R.D., Tan A.H.Y., Tu W.J., Wu F., Wagstaff K.M., Zafar A., Ali S., Diwakar H., Dahlstrom J.E. (2022). Selective targeting of protein kinase c (pkc)-θ nuclear translocation reduces mesenchymal gene signatures and reinvigorates dysfunctional cd8(+) t cells in immunotherapy-resistant and metastatic cancers. Cancers.

[156] Zheng S., Liu Y. (2024). Progress in the study of fra-2 in respiratory diseases. Int. J. Mol. Sci..

[157] Glover J.N., Harrison S.C. (1995). Crystal structure of the heterodimeric bzip transcription factor c-fos-c-jun bound to DNA. Nature.

